# Fragipan Horizon Fragmentation in Slaking Experiments with Amendment Materials and Ryegrass Root Tissue Extracts

**DOI:** 10.1155/2014/276892

**Published:** 2014-09-02

**Authors:** A. D. Karathanasis, L. W. Murdock, C. J. Matocha, J. Grove, Y. L. Thompson

**Affiliations:** Department of Plant & Soil Sciences, University of Kentucky, Lexington, KY 40546, USA

## Abstract

Slaking experiments were conducted of fragipan clods immersed in solutions of poultry manure, aerobically digested biosolid waste (ADB), fluidized bed combustion byproduct (FBC), D-H_2_O, CaCO_3_, NaF, Na-hexa-metaphosphate, and ryegrass root biomass. The fragipan clods were sampled from the Btx horizon of an Oxyaquic Fragiudalf in Kentucky. Wet sieving aggregate analysis showed significantly better fragmentation in the NaF, Na-hexa-metaphosphate, and ryegrass root solutions with a mean weight diameter range of 15.5–18.8 mm compared to the 44.2–47.9 mm of the poultry manure, ADB, and FBC treatments. Dissolved Si, Al, Fe, and Mn levels released in solution were ambiguous. The poor efficiency of the poultry manure, ADB, and FBC treatments was attributed to their high ionic strength, while the high efficiency of the NaF, Na-hexa-metaphosphate, and rye grass root solutions to their high sodium soluble ratio (SSR). A slaking mechanism is proposed suggesting that aqueous solutions with high SSR penetrate faster into the fragipan capillaries and generate the critical swelling pressure and shearing stress required to rupture the fragipan into several fragments. Additional fragmentation occurs in a followup stage during which potential Si, Al, Fe, and Mn binding agents may be released into solution. Field experiments testing these findings are in progress.

## 1. Introduction

Fragipans are naturally causing restrictive layers occurring in about 53,000 km^2^ of Kentucky soils and more than 970,000 km^2^ in the US [1, 2]. They usually form as a result of weathering of primary minerals (mainly feldspars) in relatively acidic environments. Upon dissolution, these minerals release high levels of Si, Al, and other cations (Fe, Mn), which, under dry conditions, form Si-enriched amorphous gels that harden into a cement-type material that binds the soil particles into large masses of impermeable prism-like structures [3–5]. The fragipan, which usually occurs at depths between 45 and 60 cm from the soil surface, greatly restricts water movement and root growth [6]. Therefore, it reduces the water holding potential in these soils to about one-half of that of many other crop producing soils [7]. It also causes saturated soil conditions in the winter and spring that result in adverse conditions to crops growing during this time [8, 9]. By far the biggest production problem for corn and soybeans grown on these soils is limited water holding capacity, which may reduce yields by at least 20–25% [10, 11].

Field evidence suggests that fragipan horizons occurring in landscape positions with high hydraulic loads show signs of considerable degradation, particularly in their upper part which is in contact with the developing perched water table [4, 5, 12]. However, these weathering effects are the result of hundreds or thousands of years with no practical significance. In laboratory experiments, slaking (breakdown of soil clods into smaller fragments on rapid wetting) of soil clods obtained from Btx horizons of two Fragiudalfs in the southern Mississippi Valley Silty Uplands occurred at significantly lower rates than clods obtained from Bt horizons of a Hapludalf [13]. However, the extent and severity of slaking were independent of particle size distribution and extractable Si or Fe. Aggregate stability tests by wet sieving of fragipan and nonfragipan soil materials obtained from two Fragiudalfs in Italy indicated a protective effect of organic matter and a correlation of aggregate destabilization with particle arrangement and porosity rather than particle size distribution [14].

Surprisingly, not many studies have explored alternative feasible management options that could accelerate fragipan degradation and remediate the problem. Physical treatments, including mechanical disturbance in combination with 2% organic matter application, somewhat lowered the bulk density of some fragipans but only for a limited time of 5 years [15]. Application of lime, sawdust, and fertilizer to back-filled trenches excavated into fragipan horizons also lowered their bulk density and significantly increased water storage capacity and crop yields for a duration of 16 years compared to similar untreated soils [16]. Other agricultural and industrial solid waste amendments have been used extensively in fragipan soils but mainly as alternative nutrient sources or structural stability contributors rather than fragipan remediation products [7, 17–20].

Most researchers have attributed loessial fragipan cementation to excess Si, Al, and Fe hydroxide precipitates binding soil particles and sealing a significant part of the fragipan pore system [4, 21–25]. Since the restrictive nature of fragipans has been attributed to Si-enriched amorphous binding agents, high pH amendments in the presence of dispersive materials (Na or Mg based) at low concentrations may facilitate their dissolution and reduce fragipan rigidity [26], thus allowing greater soil depths of rooting and water storage capacity. Rhoton et al. [27] evaluated under laboratory conditions a fluidized bed combustion byproduct (FBC) of pH 12 as a potential amendment to dissolve the cementing agents and decrease fragipan strength. Although different application rates did not significantly reduce fragipan strength below that of natural unamended samples, it was found that higher application rates slightly lowered fragipan strength apparently due to the dispersive effect of high Mg levels present in the FBC. It was also suggested that the true potential of fly ash as a fragipan weathering amendment may have been hindered by reprecipitation of poorly crystalline Mg-silicate phases in the closed laboratory system, while it may be more effective under natural field conditions, where dissolution products may be readily leached from the soil.

The use of cover crops has grown in popularity as means to control erosion and improve nutrient and water storage capacity, particularly in soils with limitations like fragipans [28–32]. Ryegrass (*Lolium perenne* L.), winter wheat (*Triticum aestivum* L.), hairy vetch (*Vicia villosa*), and other cover crops are most commonly used, but their effect on fragipan weathering is little understood [31–33]. Field trials by the University of Illinois suggested greater root penetration into the upper part of the fragipan by annual ryegrass plants and formation of root channels (macropores) that the succeeding spring crop can utilize to reach moisture within the cemented layer that normally is not available [34]. Planting annual ryegrass for two years allowed for 10–15 cm root penetration into the pan and improved moisture accessibility, something that corn and soybean roots cannot do by themselves. Whether this effect is exclusively physical/mechanical or it is facilitated by biochemical interactions through biomass accumulation and/or exudate excretion is unknown [20, 35–37].

The objectives of this study were to evaluate under laboratory conditions a number of amendment type materials and cover crop biomass extracts in hopes of finding effective means to accelerate the degradation of fragipan soil materials. The ultimate goal of the research findings is to test some of the most promising amendment materials and cover crops on field trials with fragipan soils.

## 2. Materials and Methods

The fragipan resistance to degradation was evaluated with slaking experiments of undisturbed fragipan clods immersed in solution extracts from amendment or cover crop biomass materials. The fragipan samples were collected from a Zanesville silt loam soil (fine-silty, mixed, active mesic, and Oxyaquic Fragiudalfs) at field moisture capacity (~−10 kPa matric potential). The undisturbed samples were sealed in plastic bags and stored under refrigeration until used. The fragipan samples were treated with solutions/extracts from the following materials: deionized water (D-H_2_O), reagent grade CaCO_3_ at solubility strength, 0.005 M NaF, 0.005 M Na-hexa-meta-phosphate, an aerobically digested biosolid waste (ADB) collected from a waste water treatment plant in Jessamine County, KY, a fluidized bed combustion byproduct (FBC) from a coal power facility, broiler litter collected from a poultry facility, and ryegrass root biomass collected from a field where it was grown as a cover crop. Poultry litter, FBC, ADB, and ryegrass root solution extracts were generated using 1 : 1 solid dry weight to D-H_2_O ratio. The ryegrass root biomass solution was extracted by 30-min boiling with D-H_2_O and cooled down to room temperature (~20°C). All solutions and extracts were filtered before interacting with the fragipan materials.

The rationale for selection of the treatments was based on the following criteria. The D-H_2_O treatment was mainly used as control. Poultry litter, CaCO_3_, and other biosolid wastes are extensively used as amendments in soils, including many with fragipan horizons. Fly ash materials have been tested before in fragipan slaking experiments with some potential benefits. Na-hexa-metaphosphate is a primary dispersing agent used in particle size analysis while NaF could serve as a dispersant and Al complexing agent. Finally, the ryegrass is widely used as a cover crop, with some recent evidence suggesting that its roots can penetrate into the upper part of the fragipan horizon.

Sections of undisturbed fragipan samples representing prism interiors from the Btx_1_ horizon were trimmed to potato shaped clods at field moisture state, approximately 8 × 5 cm in size, weighing about 140 g, and placed in 1 liter wide mouth glass jars. Each fragipan clod was immersed in 500 mL of solution extract representing the eight amendment materials using five replicates from each treatment. The jars were left undisturbed except for being subjected to a 2 min sonication treatment twice a week to accelerate degradation. Ten mL aliquots from the solutions/extracts in contact with the fragipan clods were collected the day after sonication and analyzed for pH, EC, Si, Al, Fe, and Mn [38]. Fragipan resistance to slaking was visually evaluated at every sampling for four weeks and at the end of the experiment through wet aggregate sieving analysis using the following sieve sizes: 50 mm, 22.4 mm, 15.9 mm, 12.5 mm, 7.9 mm, 6.4 mm, 4.0 mm, and 2.00 mm [39, 40]. The % contribution of each aggregate size fraction was used to calculate the aggregate MWD. Fragipan subsamples were processed for particle size distribution, pH, exchangeable cations, CEC, organic matter, and mineralogical composition [38]. Particle size distribution, selected chemical, and mineralogical characterization data for the soil studied are shown in Table 1. Statistical comparisons of treatments were performed using ANOVA (Fisher's protected least significant difference test and Duncan's test) in SAS 9.3 with *α* < 0.05 probability levels. Significant statistical relationships between slaking solution variables and aggregate MWD were determined with single and multiple regression analysis in SAS 9.3 with *α* < 0.05 (∗) and *α* < 0.01 (∗∗) probability levels.

## 3. Results and Discussion

### 3.1. Aggregate Stability Measurements

**Figure 1:**
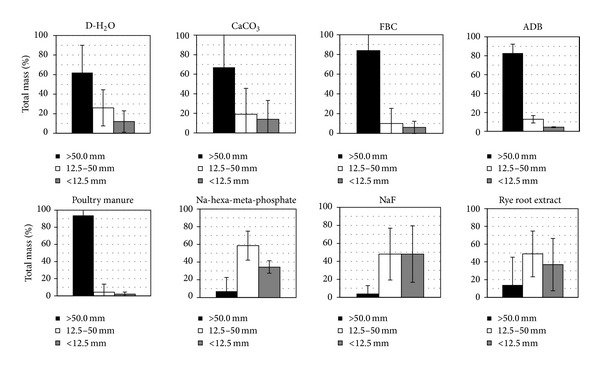
Wet aggregate size distribution of fragipan fragments in slaking experiments with D-H_2_O, CaCO_3_, FBC, ADB, poultry manure, 0.05 M Na-hexa-metaphosphate, 0.05 M NaF, and rye root extracts.

Wet sieving analysis of aggregate size distribution following completion of the experiment indicated considerable variability between and within treatments (Figure 1). Three aggregate size ranges were selected for comparison purposes including the >50 mm, the 50–12.5 mm, and the <12.5 mm fraction. The poultry manure, FBC, and ADB treatments contained the highest overall % of fragments in the >50 mm fraction with mean values of 93.8 ± 10.7, 84.0 ± 20.4, and 82.8 ± 18.2%, respectively (Figure 1). Several of these samples remained intact without any signs of alteration by the end of the experiment, while the remaining clods experienced very little degradation. Mean % values for the 50–12.5 mm size fraction for the same amendments ranged from 4.2 to 12.8 and for the <12.5 mm fraction 2.0 to 4.0. Fragipan clods exhibited intermediate resistance to D-H_2_O and CaCO_3_ treatments, with mean % values of fragments in the >50 mm size fraction of 62.0 ± 28.0 and 67.0 ± 47.2, respectively (Figure 1). In these treatments, significant fragipan degradation signs were observed only after the second week of the experiment with high standard error (SE) within treatments. Mean % values for the 50–12.5 mm size fraction ranged from 19.0 ± 26.6 for the CaCO_3_ to 26.0 ± 18.5 for the D-H_2_O treatments, and for the <12.5 mm fraction 14.0 ± 19.2 and 12.0 ± 11.0, respectively. The NaF, Na-hexa-metaphosphate, and ryegrass root treatments were the most effective in causing significant slaking in the fragipan clods (Figure 1). The degradation effects were evident before the end of the first week of the experiments. Only one replicate of five in each one of these treatments contained aggregates in the >50 mm size fraction and in all cases the original clod was fragmented. Mean % values for the >50 mm size fraction ranged from 4.0 ± 8.9 for the NaF to 14.0 ± 31.9 for the ryegrass root treatments (Figure 1). The respective values for the 50–12.5 mm size fraction were between 48.8 ± 28.5 and 58.6 ± 13.3, while for the <12.5 mm size fraction 34.4 ± 7.1 and 48.0 ± 31.3. The highest values of the smallest aggregate size fraction were observed with the NaF and the lowest with the Na-hexa-meta-phosphate treatment.

**Figure 2:**
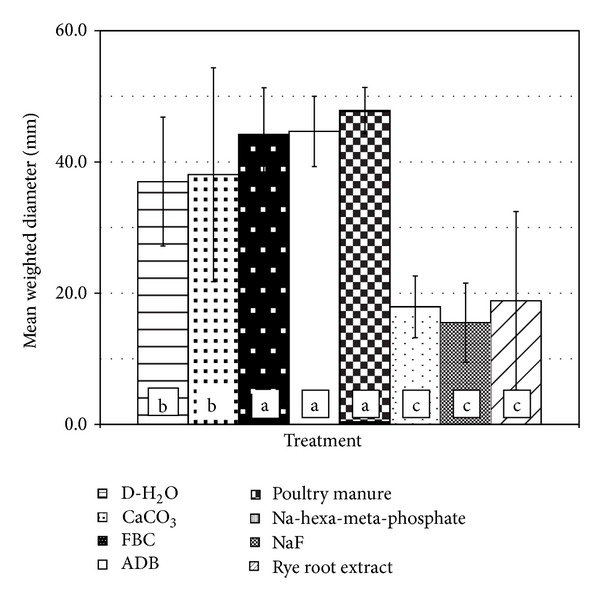
Mean weighted diameter (MWD) ± SE of fragipan fragments following 30-day slaking experiments with eight different amendment solutions/extracts (different letters designate significant differences in MWD based on Duncan's and LSD tests at *α* < 0.05).

Mean weight diameter (MWD) estimates for the eight treatments are shown in Figure 2. Statistical analysis of mean comparisons using the LSD and Duncan's tests confirmed significant differences (*α* < 0.05) between treatment groups, consistent with the trends observed in aggregate size distribution patterns shown in Figure 1. The most effective fragipan slaking treatments appeared to be those with NaF, Na-hexa-meta-phosphate, and ryegrass root solutions/extracts as shown by the smallest overall MWD size range (*α* < 0.05). The respective values ranged from 15.5 ± 6.0 for NaF to 18.8 ± 13.6 mm for the ryegrass root extract treatment. The poultry manure, ADB, and FBC treatments were the least effective in slaking the fragipan materials with MWD of 47.9 ± 3.5, 44.7 ± 5.4, and 44.2 ± 7.1 mm, respectively. These treatments also consistently showed the lowest overall variability within replicates. Fragipan clods in the presence of D-H_2_O and CaCO_3_ solutions exhibited intermediate but only slightly lower resistance to slaking compared to the poultry manure, ADB, and FBC treatments (*α* < 0.05), with MWD ranging from 37.0 ± 9.8 to 38.1 ± 16.3 mm, respectively.

### 3.2. Solution Compositions

**Figure 3:**
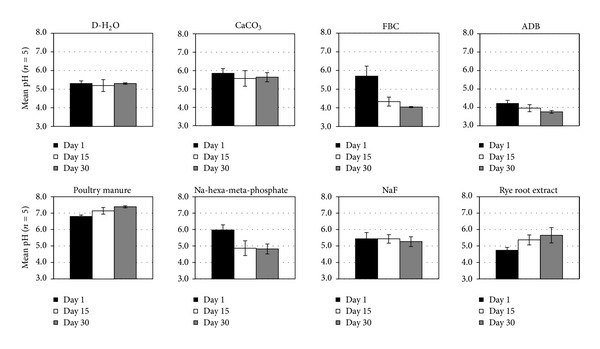
Mean ± SE solution pH levels at the beginning, the end, and the midpoint of the fragipan slaking experiments with the eight amendment treatments.

**Figure 4:**
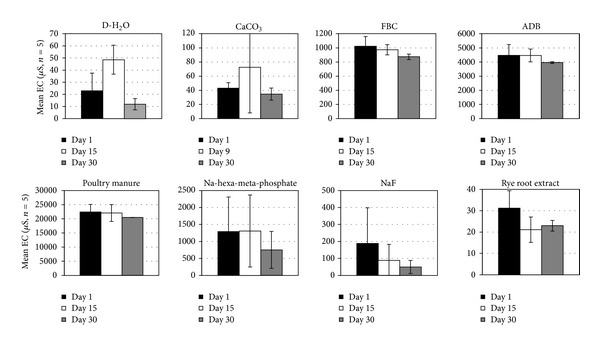
Mean ± SE solution EC levels at the beginning, the end, and the midpoint (or time of maximum level attained) of the fragipan slaking experiments with the eight amendment treatments.

**Figure 5:**
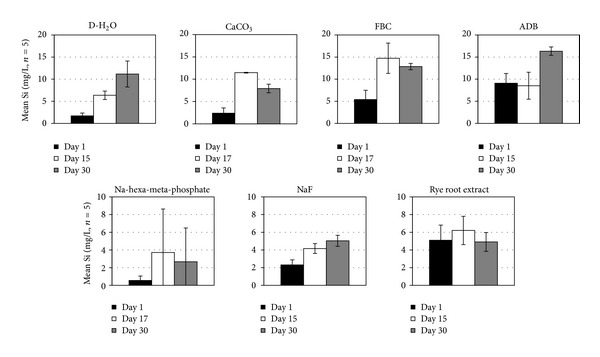
Mean ± SE solution Si concentrations at the beginning, the end, and the midpoint (or time of maximum level attained) of the fragipan slaking experiments with the eight amendment treatments.

**Figure 6:**
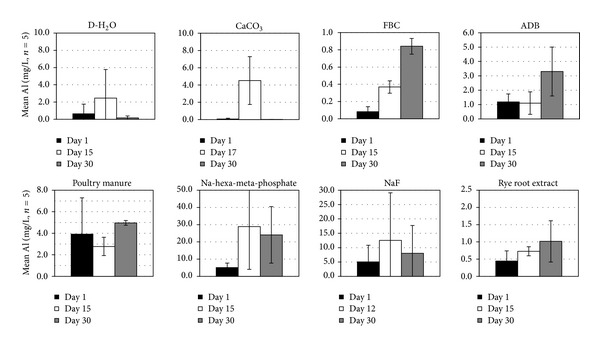
Mean ± SE solution Al concentrations at the beginning, the end, and the midpoint (or time of maximum level attained) of the fragipan slaking experiments with the eight amendment treatments.

**Figure 7:**
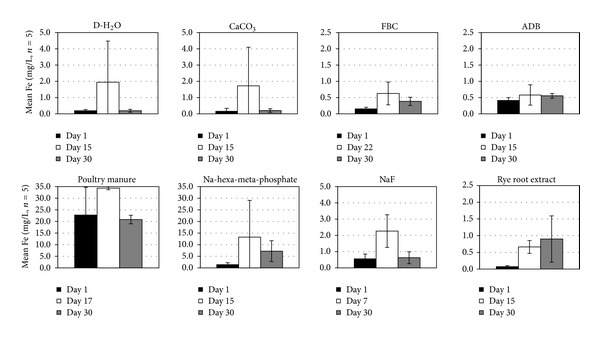
Mean ± SE solution Fe concentrations at the beginning, the end, and the midpoint (or time of maximum level attained) of the fragipan slaking experiments with the eight amendment treatments.

**Figure 8:**
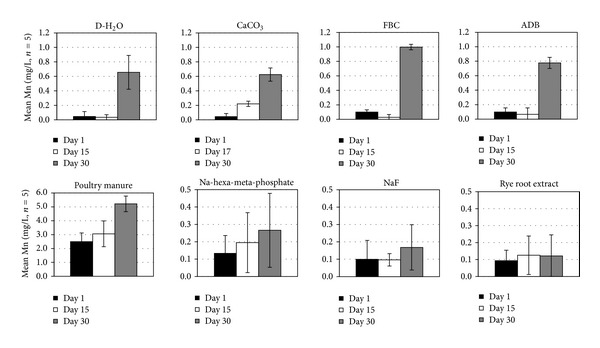
Mean ± SE solution Mn concentrations at the beginning, the end, and the midpoint (or time of maximum level attained) of the fragipan slaking experiments with the eight amendment treatments.

Concentrations of selected soluble components in contact with the fragipan clods over the length of the experiments are shown in Figures 3–8. The plotted values represent the initial, final, and middle time compositions unless a maximum or minimum occurred during the experiment. Solution mean pH values ranged from 3.8 in the ADB to 7.2 in the poultry manure treatments (Figure 3). Statistically significant trends (*α* < 0.05) of mean solution pH levels over the length of the experiment among treatments followed the sequence PM > CaCO_3_ > NaF = rye root extract = D-H_2_O = Na-hexa-meta-phosphate > FBC > ADB. The pH values remained relatively constant throughout the experiment between 5.2 and 5.8 in the D-H_2_O, CaCO_3_, and NaF treatments. The more drastic changes occurred in the FBC and Na-hexa-metaphosphate treatments, where solution pH dropped from around 6.0 at the beginning of the experiment to 4.0 and 4.8, respectively, indicating significant hydrolysis effects, probably from Al released into solution. The rest of the solutions experienced only minor changes over time, with slight increases in the poultry manure and ryegrass treatments or a slight decrease in the ADB treatment. Even though the poultry manure had the highest solution pH, it showed the lowest overall fragipan slaking potential among the treatments, suggesting that the higher pH was not enough to induce greater dispersion.

Mean solution EC values differed drastically among treatments, being lowest (<70 *μ*S/cm) in the ryegrass root, D-H_2_O, and CaCO_3_ solutions and highest (4,000–22,000 *μ*S/cm) in the ADB and poultry manure treatments (Figure 4). Statistically significant trends (*α* < 0.05) of mean EC solution values among treatments over the length of the experiment followed the sequence PM > ADB > Na-hexa-meta-phosphate = FBC > NaF > CaCO_3_ > D-H_2_O = rye root extract. Even though EC values decreased slightly over time, they likely contributed to fragipan resistance to slaking by inhibiting dispersibility [40]. A similar inhibition may have occurred in the FBC solution where EC values ranged from around 1000 to 840 *μ*S/cm and the pH dropped to 4.0 during the experiment. Mean solution EC values reached maxima at or before the midpoint of the experiment in the D-H_2_O and the CaCO_3_ treatments before reaching the lowest level at the end of the experiment, probably representing a release stage of soluble components followed by a resorption or reprecipitation stage. Decreasing trends in solution EC values were also exhibited by the Na-hexa-meta-phosphate, NaF, and ryegrass root treatments, suggesting a partial reprecipitation or resorption of earlier solubilized fragipan constituents by the relatively large amount of finer aggregates/particles produced during the slaking process.

Mean concentrations of soluble Si released in solution for the different treatments over the course of the experiment are shown in Figure 5. Since Si is assumed to be a main contributor in the cementation of fragipan, higher Si releases were expected to correspond with greater slaking efficiency trends. However, since each amendment solution used had different inherent soluble Si levels at the beginning of the experiment, comparisons accounted only for differences between initial and final or initial and maximum concentrations. Statistically significant trends (*α* ≤ 0.05) of mean Si concentrations in solution among treatments for the duration of the experiment followed the sequence ADB > FBC > CaCO_3_ = D-H_2_O = rye root extract > NaF > Na-hexa-meta-phosphate. The highest amounts of Si released into solution (8–10 mg/L) during the slaking experiments were associated with the D-H_2_O, CaCO_3_, FBC, and ADB treatments (Figure 5). These concentrations increased regularly over time for the D-H_2_O, and ADB treatments, reaching maximum levels at the conclusion of the experiment, showed a decreasing trend with time in the CaCO_3_ and FCB treatments, apparently due to reprecipitation. An increasing Si concentration trend with time was also observed with the NaF treatment, but the total amount of Si released was <3 mg/L. The Na-hexa-meta-phosphate and ryegrass root treatments showed Si release maxima around the midpoint of the experiment in the range of 1–3 mg/L before a final drop at the end of the experiment. Soluble Si release was not measured in the poultry manure treatment due to interference with the dissolved organic matter. Surprisingly, the three most effective fragipan slaking treatments (NaF, Na-hexa-metaphosphate, and ryegrass root) as documented by the wet aggregate analysis measurements released the lowest levels of soluble Si levels, suggesting that additional mechanisms may be involved in promoting the slaking of fragipan materials [14].

Mean concentrations of Al, which is another component that along with Si presumably contributes to fragipan rigidity, ranged from <1 to about 25 mg/L, with the highest levels associated with the Na-hexa-meta-phosphate and NaF treatments (Figure 6). Ryegrass root, FBC, and poultry manure treatments had the lowest amounts of Al release. Statistically significant trends (*α* < 0.05) among treatments of mean Al concentrations in solution for the duration of the experiment followed the sequence Na-hexa-meta-phosphate > NaF > PM = ADB = D-H_2_O = CaCO_3_ = rye root extract = FBC. The D-H_2_O and CaCO_3_ treatments showed moderate concentrations that peaked around the midpoint and dropped sharply to near 0 at the end of the experiment, suggesting reprecipitation or resorption processes. Comparing both Si and Al release among treatments, it appears that NaF and Na-hexa-meta-phosphate treatments were most consistent in maintaining moderate to high soluble levels of both by the end of the experiment even though some of the released Al may have been sequestered by formation of AlF complexes or Al-phosphates [41].

Mean soluble Fe levels released during the slaking experiments ranged from <1 to about 12 mg/L (Figure 7). The highest values were observed in the Na-hexa-meta-phosphate and poultry manure solutions and the lowest with the ADB, FBC, and ryegrass root treatments. Statistically significant trends (*α* < 0.05) among treatments of mean Fe concentrations in solution for the length of the experiment followed the sequence PM > Na-hexa-meta-phosphate > CaCO_3_ = D-H_2_O = NaF = rye root extract =ADB = FBC. In most cases (except for the ryegrass root and the ADB treatments) maximum levels of Fe release occurred before the end of the experiment, suggesting the occurrence of resorption or reprecipitation reactions in later stages of treatment. The high levels of Fe released in the poultry solution were more likely the result of anoxic Fe dissolution from organic particles or the surface of the fragipan clods, since very little or no slaking occurred during the treatment [42, 43]. Mean Mn concentrations released in solution were generally low compared to other solution components (<1 to 2.5 mg/L), reaching maximum or near maximum values in the end of the experiment (Figure 8). Significantly higher (*α* < 0.05) Mn concentrations occurred in the poultry manure solution than in the other treatments, where anoxic conditions were more prevalent [43]. Since no or minimum fragipan slaking occurred with poultry manure treatments, most of the Mn released was probably associated with organic particulate material or the surface of the fragipan clods, thus minimizing its potential contribution as a binding component.

Mean concentrations of soluble Ca, Mg, K, and Na in the end of the experiment are shown in Table 2. The ADB, poultry manure, and FBC treatments had significantly higher (*α* < 0.05) Ca (50–276 mg/L) and Mg (17–77 mg/L) levels in solution. The rest of the treatments averaged <2 mg/L Ca and <10 mg/L Mg, with values near 0 for D-H_2_O, CaCO_3_, and NaF solutions. The poultry manure had by far the highest (*α* < 0.05) soluble K levels (1853 mg/L), while the D-H_2_O, CaCO_3_, and NaF solutions the lowest (<0.5 mg/L). The highest Na concentrations (*α* < 0.05) were observed in the NaF, Na-hexa-metaphosphate, and poultry manure solutions (493–1056 mg/L) and the lowest with the D-H_2_O and CaCO_3_ treatments (<1.8 mg/L). A soluble sodium ratio (SSR) function equal to soluble Na/soluble (Ca + Mg + K) was also estimated to further explore the dispersive characteristics of Na relative to other ions since the three weakest slaking treatments (poultry manure, ADB, and FBC) showed the lowest overall (*α* < 0.05) SSR values (Table 2).

### 3.3. Statistical Relationships

There was no significant relationship between final solution pH and MWD whether considering the entire population of samples used in all treatments or after grouping the samples by treatments showing statistically similar MWD trends. Apparently, either the pH range used in the experiment was not broad enough to significantly impact aggregate size and stability or the effect was counterbalanced by opposite trends within the same MWD group. A significant relationship (*R* ^2^ = 0.25*) was observed between EC and MWD with a tendency for less slaking and larger aggregate size with increasing EC. This is consistent with literature findings that suggest increasing trends of aggregate stabilization at high electrolyte concentrations [44, 45]. This relationship was probably enhanced by the high EC values of the poultry manure, ADB, and FBC solutions which produced the least slaking and the largest size aggregates. A significant relationship was also observed between MWD and Si released in solution (*R* ^2^ = 0.37**). However, the positive trend between these two variables suggesting higher soluble Si concentrations being associated with lower slaking effects is somewhat surprising. Assuming that the amount of Si released is the result of fragipan degradation, a negative correlation would have been more fitting. However, it is not possible to distinguish between Si released as a result of fragipan matrix breakdown or dissolution from the surface of still intact fragipan clods. Apparently, most of the Si released in the least effective slaking treatments (FBC, ADB, and poultry manure) was associated with the fragipan clod surfaces rather than the interior binding agents and may have been induced by interaction with soluble organics. Indeed, the presence of organic acids has been documented to significantly increase the solubility of siliceous materials through formation of organic complexes [46]. Although this association may have obscured the soluble Si-MWD relationship in the most effective slaking treatments that released low to moderate Si levels, additional slaking mechanisms should not be discounted.

Weak positive trends were also present between MWD and soluble Al (*R* ^2^ = 0.15*) and Mn (*R* ^2^ = 0.13*), but this relationship was also skewed by the high levels of dissolved organic carbon in treatments with low slaking efficiency that caused increased soluble Mn release from the surface of the clods. Soluble Fe released in solution during the experiment did not show significant correlations with MWD of the fragipan aggregates, implying that its role as a binding agent affecting fragipan resistance to slaking in these experiments was limited or the effect was overshadowed by fragipan surface clod solubility reactions induced by soluble organics. Considering that none of the hypothesized binding agents (Si, Al, Fe, and Mn) have shown the expected statistical trends, it is likely that the slaking of the fragipan clods has been induced by alternative mechanisms not necessarily depending on the dissolution of these components.

The positive correlations of Ca (*R* ^2^ = 0.22*), Mg (*R* ^2^ = 0.23*), and K (*R* ^2^ = 0.13*) in solution with MWD suggested that their role in the slaking process was mostly inhibitive rather than conducive with increasing concentrations [47]. Therefore, low levels of these components in solution and low ionic strength (low EC values) are important for generating a dispersive environment that could weaken fragipan resistance to slaking [48]. Indeed, the treatments showing moderate to high slaking efficiency had low solution EC levels, while the least effective treatments had some of the highest. The importance of a dispersive environment in enhancing the slaking process was demonstrated by the overall negative relationship between MWD and soluble Na (*R* ^2^ = 0.20*), which was improved to *R* ^2^ = 0.40** when using the SSR function. This underscores the role of Na in excess of other ions in creating an environment conducive to weakening fragipan resistance regardless of the slaking mechanism involved [48]. A multiple regression analysis model including soluble Si and SSR with opposing trends accounted for 52% of the variation (*α* < 0.05) in the MWD of the slaked fragments. Only the addition of soluble K significantly improved the predictability of the model (*R* ^2^ = 70, *α* < 0.05). Potassium has been reported to exhibit dispersive properties in association with Na in low ionic strength environments [49, 50].

Assuming that the role of binding agents (Si, Al, Fe, and Mn) through dissolution reactions in the slaking process is ambiguous in these experiments, the involvement of Na may be more significant than originally perceived. Soil hydration reactions proceed at a much faster rate in the presence of dilute electrolytes than at higher concentrations. Therefore, the high ionic strength of poultry manure, ADB, and FBC solutions considerably slowed the hydration of fragipan clods. Elevated electrolyte concentrations usually cause increases in the surface tension of water molecules and larger contact angles with the solid surface [44, 49, 51]. Some of these contact angles may have even increased further by organic films coating the surface of fragipan clods in treatments with high levels of dissolved organics (poultry manure, ADB), thus forming a less hydrophilic surface for water penetration [47, 52, 53]. Water infiltration into soil micropores is enhanced when the contact angle of the hydrated ions is relatively small. The contact angle of hydrated Na ions in relatively low ionic strength but high SSR solutions being smaller than that of Ca and Mg will expedite fragipan micropore penetration and increase capillary rise [51, 54]. That can generate a swelling pressure inside the capillaries which causes the entrapped air to implode the fragipan clod along planes of weakness [45, 55, 56]. This is supported by the very small fraction of aggregates below the 2 mm size range, indicating limited dispersion of the fragipan material. Hydrated Ca and Mg ions as well as high electrolyte concentration solutions with low SSR having slower penetration within the capillary space of the fragipan clod allow more time for air diffusion in the water, which reduces the pressure on the matrix and allows it to maintain its original cohesiveness. Similar effects on soil aggregate stability by Na and Ca electrolytes of different strength and SAR values were observed by Abu-Sharar et al. [48] and were attributed to repulsion forces developed within the capillaries that allowed increased shearing stresses to break down the aggregates without significant clay dispersion. Cass and Sumner [57] also reported a random breakdown of soil aggregates along planes of weakness within the matrix of the aggregate rather than at the periphery prior to clay dispersion from the rupturing effect of internal swelling pressure or from shearing stresses in the presence of Na electrolytes.

### 3.4. Proposed Slaking Mechanism

Based on this evidence, it is likely that the slaking mechanism of the fragipan clods in our experiments may involve at least two steps. During the first step, fragipan fragmentation occurs with the dispersive action of Na in high SSR solutions, increased capillary rise within the clod matrix, and a swelling pressure build-up that increases shearing stress and causes the rupture of the clod into several fragments [44, 45, 58]. Presence of entrapped air bubbles within the capillaries may accentuate the fragmentation process by building extra pressure and inducing implosion of the clods [53, 56, 58, 59]. The initial fragmentation stage appears to be mainly physical but it may be followed by additional fragmentation steps into smaller aggregates during which physical and possibly chemical dissolution reactions may be involved. During this stage a small number of binding agents (Si, Al, Fe, and Mn) exposed on the surface of broken fragments may be released into solution and accelerate the degradation. Given the low solubility and the slow dissolution kinetics of these agents, a certain amount of the solubilized fraction may reprecipitate or resorb as polymers on the exposed surfaces if the degradation products are not flushed out of the system. In an open soil environment, leaching of these dissolved components away from the fragipan matrix will likely promote further dissolution and expedite fragipan fragmentation. In solutions of high electrolyte concentration with an abundance of Ca or Mg ions, the first stage of fragmentation is inhibited due to less and slower capillary penetration and lower swelling pressure that does not build the critical shearing stress needed to cause meaningful fragipan fragmentation [45]. The slower pace of water movement within the capillaries also allows enough time for the entrapped air to diffuse in the water thus eliminating the build-up of additional pressure on the capillary walls. Since minimal or no fragmentation occurs in these treatments, most of the Si, Al, Fe, or Mn released during the experiments was dissolved from the periphery of the fragipan clods and not from the interior of the fragments. This release may be enhanced by dissolved organic carbon (DOC) in solutions of organic amendments but organic coatings of fragipan surfaces may also inhibit water penetration in the capillaries by creating a semihydrophobic barrier.

## 4. Conclusions

The findings of these experiments suggested that amendment materials producing solutions with relatively low ionic strength and elevated SSR (NaF, Na-hexa-meta-phosphate, and ryegrass root) create an environment more conducive to fragipan slaking than that of high electrolyte concentrations, Ca, Mg, or DOC enriched solutions (poultry manure, ADB, and FBC). The facilitation of water entry into fragipan capillaries in the presence of Na ions generates the critical swelling pressure and shearing stress to cause fragipan fragmentation. Additional fragmentation with minimal clay dispersion occurs in a follow-up stage during which a moderate number of potential binding agents (Si, Al, Fe, and Mn) are released in solution. These dissolved constituents may polymerize in situ and resorb on the surfaces of the broken fragments if not flushed out of the system. The results suggest that selecting an amendment with low salt content and elevated SSR may produce favorable conditions for fragipan degradation. Currently, we are exploring the potential effects of other Na-based amendments on fragipan slaking before testing these findings under field conditions, which will be the next phase of this project. A potential incorporation of a wetting agent (surfactant) in the applied amendment may facilitate the fragipan fragmentation process.

## Figures and Tables

**Table 1:** Selected physical, chemical, and mineralogical properties of the Zanesville soil used in the study.

Horizon	Depth (cm)	Sand (%)	Silt (%)	Clay (%)	Texture	pH (1 : 1 H_2_O)	Ca	Mg	K	Na	CEC	O M (%)	Clay mineralogy^†^
							meq/100 g				
Ap	0–20	8.7	70.1	21.2	Silt loam	7.52	4.98	1.07	0.13	0.05	9.0	3.94	V/HIV44 K26 Mi9 Q10 Gi8 F3
Bt_1_	20–42	5.3	75.4	19.3	Silt loam	5.05	3.12	1.58	0.16	0.08	12.7	2.10	V/HIV52 K23 Mi9 Q9 Gi3 F4
Bt_2_	42–65	4.2	72.4	23.4	Silt loam	4.52	1.42	2.38	0.17	0.15	13.0	0.33	Sm23 V/HIV31 K24 Mi11Q7 F4
Btx_1_	65–127	10.5	65.3	24.2	Silt loam	4.28	0.80	2.96	0.13	0.18	11.8	0.19	Sm23 V/HIV40 K28 Q5 F4
Btx_2_	127–140+	13.9	55.4	30.7	Silty clay loam	4.23	1.05	4.21	0.10	0.30	13.3	0.19	Sm23 V/HIV18 K39 Q9 Gi4 F3 Go4

^†^Sm: smectite; V/HIV: hydroxy-interlayered vermiculite; K: kaolinite; Mi: mica; Q: quartz; Gi: gibbsite; F: feldspar; Go: goethite.

**Table 2:** Mean ± SE solution concentrations of Ca, Mg, K, Na, and SSR in the fragipan slaking experiments with the eight amendment treatments over the 30-day experimental period.

Treatment	Ca	K	Mg	Na	SSR
	mg L^−1^			
D-H_2_O	0.16 ± 0.08	0.17 ± 0.16	0.01 ± 0.01	0.52 ± 0.17	0.603
CaCO_3_	0.13 ± 0.05	0.16 ± 0.18	0.01 ± 0.02	1.77 ± 0.88	0.851
FBC	49.95 ± 5.52	0.63 ± 0.10	17.63 ± 2.10	4.94 ± 0.08	0.068
ADB	276.25 ± 7.55	12.75 ± 0.42	76.95 ± 2.55	39.33 ± 3.28	0.097
Poultry manure	73.33 ± 1.89	1852.66 ± 18.27	37.76 ± 1.08	493.0 ± 7.21	0.201
Na-hexa-meta-phosphate	2.16 ± 1.99	0.38 ± 0.02	9.41 ± 8.63	881.90 ± 6.73	0.987
NaF	0.10 ± 0.06	0.15 ± 0.07	0.08 ± 0.03	1055.77 ± 8.40	0.999
Rye root extract	0.67 ± 0.63	3.61 ± 3.08	0.57 ± 0.55	8.03 ± 7.33	0.623
